# One Step before 3D Printing—Evaluation of Imaging Software Accuracy for 3-Dimensional Analysis of the Mandible: A Comparative Study Using a Surface-to-Surface Matching Technique

**DOI:** 10.3390/ma13122798

**Published:** 2020-06-21

**Authors:** Antonino Lo Giudice, Vincenzo Ronsivalle, Cristina Grippaudo, Alessandra Lucchese, Simone Muraglie, Manuel O. Lagravère, Gaetano Isola

**Affiliations:** 1 Department of General Surgery and Surgical-Medical Specialties, School of Dentistry, University of Catania, Policlinico Universitario “Vittorio Emanuele—G. Rodolico”, Via S. Sofia 78, 95123 Catania, Italy; nino.logiudice@gmail.com (A.L.G.); vincenzo.ronsivalle@hotmail.it (V.R.); simonemuraglie@live.it (S.M.); 2 Department of Orthodontics, University of Sacred Heart of Rome, 00168 Rome, Italy; Cristina.Grippaudo@unicatt.it; 3 Department of Orthodontics, Vita-Salute San Raffaele University, 10,090 Milan, Italy; lucchese.alessandra@unisr.it; 4 Orthodontic Graduate Program, ECHA 5-524, Faculty of Medicine and Dentistry, University of Alberta, 11405-87 Ave, Edmonton, AB T6G1Z1, Canada; mlagravere@ualberta.ca

**Keywords:** dental 3D scanner, dental 3D rendering, printing, segmentation, accuracy, scanner, 3D biomaterials, manufacturing, dentistry, models

## Abstract

The accuracy of 3D reconstructions of the craniomaxillofacial region using cone beam computed tomography (CBCT) is important for the morphological evaluation of specific anatomical structures. Moreover, an accurate segmentation process is fundamental for the physical reconstruction of the anatomy (3D printing) when a preliminary simulation of the therapy is required. In this regard, the objective of this study is to evaluate the accuracy of four different types of software for the semiautomatic segmentation of the mandibular jaw compared to manual segmentation, used as a gold standard. Twenty cone beam computed tomography (CBCT) with a manual approach (Mimics) and a semi-automatic approach (Invesalius, ITK-Snap, Dolphin 3D, Slicer 3D) were selected for the segmentation of the mandible in the present study. The accuracy of semi-automatic segmentation was evaluated: (1) by comparing the mandibular volumes obtained with semi-automatic 3D rendering and manual segmentation and (2) by deviation analysis between the two mandibular models. An analysis of variance (ANOVA) was used to evaluate differences in mandibular volumetric recordings and for a deviation analysis among the different software types used. Linear regression was also performed between manual and semi-automatic methods. No significant differences were found in the total volumes among the obtained 3D mandibular models (Mimics = 40.85 cm^3^, ITK-Snap = 40.81 cm^3^, Invesalius = 40.04 cm^3^, Dolphin 3D = 42.03 cm^3^, Slicer 3D = 40.58 cm^3^). High correlations were found between the semi-automatic segmentation and manual segmentation approach, with R coefficients ranging from 0,960 to 0,992. According to the deviation analysis, the mandibular models obtained with ITK-Snap showed the highest matching percentage (Tolerance A = 88.44%, Tolerance B = 97.30%), while those obtained with Dolphin 3D showed the lowest matching percentage (Tolerance A = 60.01%, Tolerance B = 87.76%) (*p* < 0.05). Colour-coded maps showed that the area of greatest mismatch between semi-automatic and manual segmentation was the condylar region and the region proximate to the dental roots. Despite the fact that the semi-automatic segmentation of the mandible showed, in general, high reliability and high correlation with the manual segmentation, caution should be taken when evaluating the morphological and dimensional characteristics of the condyles either on CBCT-derived digital models or physical models (3D printing).

## 1. Introduction

Cone beam computed tomography (CBCT) represents the three-dimensional (3D) imaging method of choice in oral and maxillofacial fields [1,2]. Although multi-slice computed tomography (MSCT) presents a higher resolution and a higher contrast-to-noise-ratio [3,4], CBCT scans offer adequate images for diagnostic purposes, at a lower risk of radiation exposure for patients [5,6,7]. In this respect, a 3D reconstruction of the craniomaxillofacial region using CBCT is employed in the diagnosis and planning of orthodontic treatment [8], maxillofacial surgery [9] and oral implantology [10], often involving the transformation of a physical object (3D printing). 3D rendering of the maxilla and mandibular jaws is extremely useful for pre-operative surgical planning and post-operative follow-up evaluation in orthognathic surgery and for the analysis of morphological, dimensional and positional changes before and after orthodontic therapies [11,12,13]. Moreover, it is fundamental to analyze and detect the magnitude and location of skeletal facial asymmetry, deformities, or asymmetry of temporomandibular joints (TMJ) and to plan surgical correction [14,15,16].

3D rendering is based on a process called segmentation, which is described as the virtual separation of the anatomical region with the removal of all other structures of noninterest for better visualization and analysis [17]. Segmentation can be performed both manually or automatically. The manual approach is user-dependent and is performed slice by slice with the software pooling all slices to reconstruct a 3D volume. Conversely, the semiautomatic segmentation is a computer-aided approach (hybrid). Here, the procedure usually starts with two user-guided interactive steps, i.e., the placement of initial seed regions in all three slices and the selection of a threshold interval (Hounsfield units), to provide information on the texture and background for the software [17,18]. Then, the software automatically selects the voxel of the interested region and excludes the surrounding structures. 

Manual segmentation represents the gold standard for the 3D rendering of maxillofacial structures (CMF) since it allows for the detection of areas with low bone density or with no well-defined boundaries due to their low contrast and proximity to other structures [3]. However, semiautomatic segmentation is faster than a manual approach, which is relevant from a clinical perspective. Furthermore, semi-automatic segmentation is not influenced by intra-operator reliability [17], which is also important for clinical and research purposes. 

The accuracy of CBCT-derived 3D segmented models can be affected by several variables such as voxel size, field of view, patient position, artifacts, and the beam inhomogeneity of CBCT scanners [3]. Additionally, the characteristics of the software used for segmentation can affect the rendering of specific craniofacial structures. In this respect, the reliability and accuracy of software for the semi-automatic segmentation of the respiratory region has been recently validated [19,20,21,22], while further studies are needed on this topic for the evaluation of the virtual anatomical segmentation of other specific CMF structures such as the mandible [22]. 

To date, the market has proposed a plethora of software for analyzing digital imaging communications in medicine (DICOM), most of them including semi-automatic segmentation tools. However, the creation of CBCT-based segmentation models is still not a common procedure in orthodontic/dental practice, since the software often presents low usability, requires higher electronic computer power and is licensed by its manufacturer, increasing the financial costs. Free-source DICOM viewers are also available online. Most of them include specific segmentation algorithms and were developed in a university setting or a small research group; as a consequence, clinicians might not be aware of such open-source options. However, no studies in the literature have assessed the accuracy of the semi-automatic segmentation method in detecting the volumetric and morphological characteristics of CMF structures—for example, the mandible—in comparison with manual segmentation, which represents the gold standard.

In this respect, the aim of the present study was to analyze the accuracy of three free-source and one licensed software in comparison with a manual segmentation approach for the semi-automatic segmentation of the mandible. For this purpose, we referred to a specific 3D digital diagnostic technology involving the surface-to-surface matching and deviation analysis [23,24] of 3D rendered mandibular models. The null hypothesis was the absence of significant differences in both the accuracy and usability of semi-automatic segmentation software compared to manual segmentation.

## 2. Materials and Methods 

The present CBCT study followed the Helsinki Declaration on medical protocols and ethics and received a positive response by the Institutional Review Board of Indiana University–Purdue University (IRB protocol number: Pro00075765). The study sample was obtained from previously published materials [25], in order to avoid unnecessary or additional radiation exposure to the patients. Because this study was an archive study, ethics committee approval was not needed. The study group included 20 subjects (9 males and 11 females, mean age 23.58 ± 3.33 years) selected from a larger sample of patients who required surgically assisted rapid maxillary expansion treatment.

All images were acquired with the KODAK 9500 3D^®^ (Carestream Health, Inc., Marne-la-Vallée, France, 90 kV, 10 mA, 0.2 mm voxel size) with the patient in maximum intercuspation and the Frankfort horizontal plane parallel to the floor [8]. The inclusion criteria were good quality CBCT scans, a field of view (FOV) including the whole mandibular bone, the absence of artifacts or image distortion, craniofacial deformities, signs or symptoms of temporomandibular disorder, third molar impaction and dental implants.

CBCT scans were preliminary imported into Dolphin 3D software (Dolphin Imaging, version 11.0, Chatsworth, CA, USA) to perform skull reorientation, according to a validated protocol [17,26]. Afterwards, the re-oriented CBCT scans were exported, and five CBCT viewer software programs were used to segment and generate a 3D rendering of the patients’ mandibular bone.

**Figure 1:**
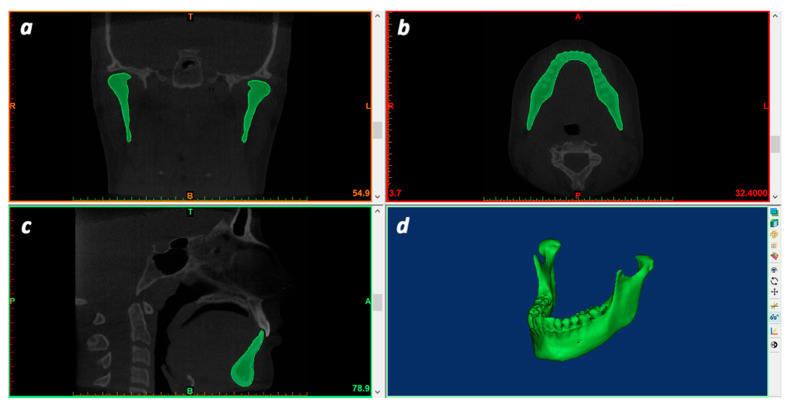
Manual segmentation of mandibular jaw (ground truth); (**a**), coronal view; (**b**), axial view; (**c**), sagittal view; (**d**), 3D rendered mandible model.

In particular, the Mimics software (version 21.0; Materialise, Leuven, Belgium) was used to perform a fully manual segmentation of the lower jaw, which served as the ground truth (gold standard) of the present investigation (Figure 1). The ground truth data for the lower jawbone boundaries were obtained by a manual slice-by-slice segmentation of the jaw data sets [27,28].

The semi-automatic segmentation of the mandible was carried out by using 4 software types, i.e., Dolphin3D (Dolphin Imaging, version 11.0, Chatsworth, CA, USA), Invesalius (version 3.0.0; Centro de Tecnologia da Informação Renato Archer, Campinas, SP, Brazil), ITK-Snap (version3.8.0; www.itksnap.org) and 3D Slicer (version 4.10.2; http://www.slicer.org). Segmentations were performed according to each software manufacturer’s recommendations and using an interactive threshold technique, which means that the operator selected the best threshold interval for visualizing the entirely of the anatomic boundaries of the mandible. Semi-automatic segmentation of the mandible was performed in this study by using the interactive threshold technique which means that operator selected the best threshold interval for visualizing the entirely of the anatomic boundaries of the mandible. Once the segmentation mask was obtained in each software, it was rendered into a 3D model and exported in STL ASCII electronic format. A detailed description of each software program is shown in Table 1. 

In order to determinate the accuracy of the semi-automatic segmentation performed with each software, a surface deviation analysis was conducted by superimposing each obtained mandibular shell with the ground truth mandibular shell (obtained from manual segmentation). Briefly, the workflow of the 3D deviation analysis is described below.

*Step 1—3D Model Superimposition and Final Registration*

**Figure 2:**
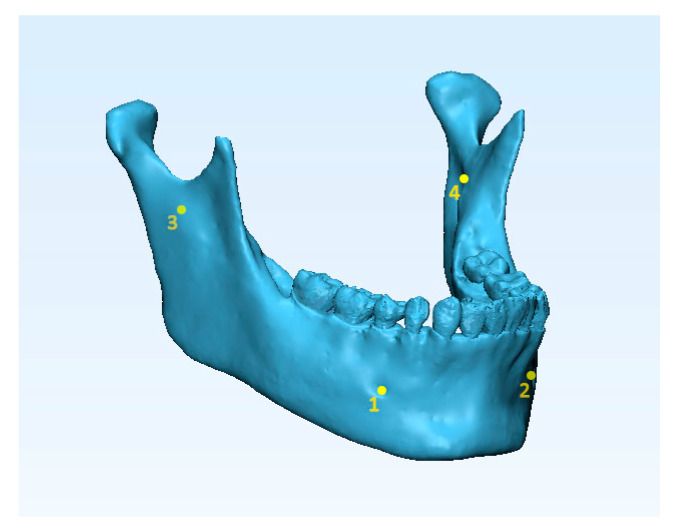
Landmarking four points on 3D mandibular model superimposition: 1–2, the geometric center of the left and right metal foramina; 3–4, left and right mandibular lingual at the inner surface of ramus.

Each mandibular model obtained from tested software (semi-automatic segmentations) was superimposed to the ground truth mandibular model (manual segmentation). A preliminary registration was carried out by selecting the same 4 points on the surface of the 3D models (Figure 2): (1–2) the geometric center of the left and right metal foramina; (3–4) the left and right mandibular lingual at the inner surface of the ramus. Then, to enhance the quality of the superimposition, a surface-based registration was made by using the ‘Best fit alignment’ function. Using the ground truth mandibular model as the reference, the final superimposition was carried out by setting the precision to at least 0.01 mm.

*Step 2—3D Models Definition (Exclusion of Teeth and Alveolar Process)*

**Figure 3:**
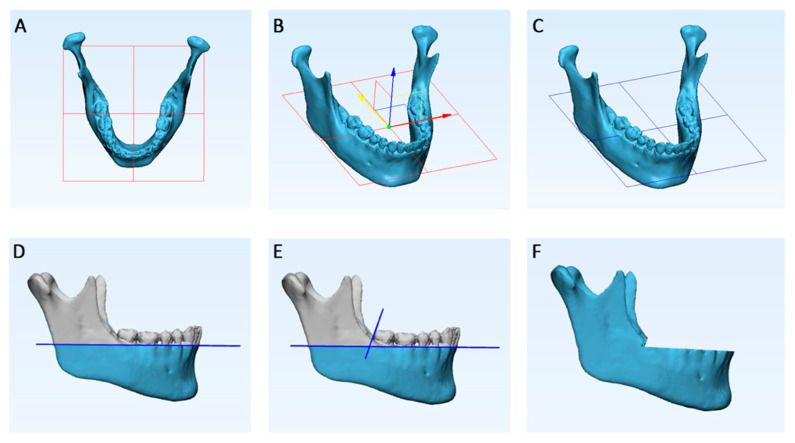
Each 3D mandibular model obtained from semi-automatic segmentation was superimposed to its ground truth model (manual segmentation) in order to reliably remove alveolar processes and teeth. (**A**), occlusal plane (OP) constructed by selecting three points, respectively, the mesio-buccal cusp tip of the mandibular first molars and the interproximal point between the two mandibular central incisors; (**B**,**C**), the OP was manually translated on the Y-axis (vertical axis) until it reached the apical position of the lowest dento-gingival junction; (**D**), sagittal view of the defined horizontal cutting plane; (**E**), construction of a distal cutting plane passing through the tangent line to distal wall of the second mandibular molars; (**F**), removal of the alveolar process according to the two defined cutting planes.

On the two superimposed models, an occlusal plane (OP) was constructed by selecting 3 points, respectively, the mesio-buccal cusp tip of the mandibular first molars and the interproximal point between the two mandibular central incisors. The OP was manually translated on the Y-axis (vertical axis) until it reached the apical position of the lowest dento-gingival junction. Finally, a distal plane passing through the tangent line to the distal wall of the second mandibular molars was created. These two cutting planes were constructed to exclude the teeth and the alveolar process from the tested mandibular shells (Figure 3).

*Step 3—3D Deviation Analysis and Assessment of Volumetric Dimension*

**Figure 4:**
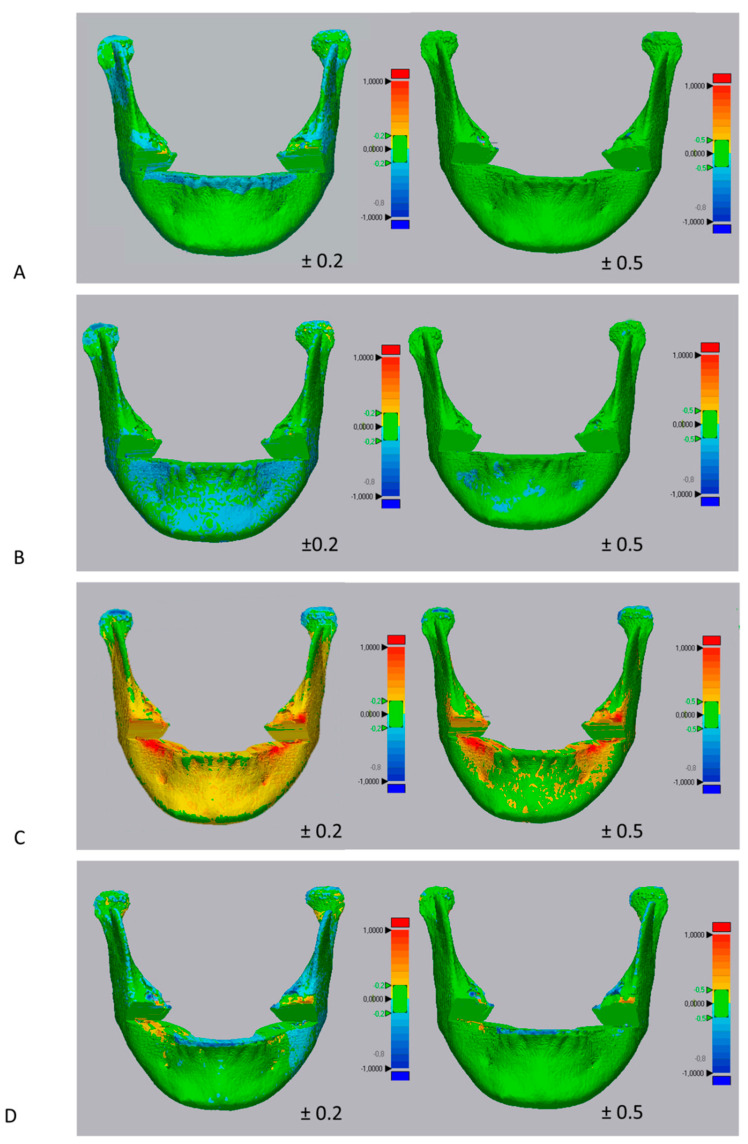
Surface-based deviation analysis between 3D mandibular models obtained with semi-automatic segmentation and its ground truth model (manual segmentation). (**A**) ITK-Snap; (**B**) Invesalius; (**C**) Dolphin 3D; (**D**) Slicer 3D. The green of the color scale bar, on the right, represents the range of tolerance. Left side—color map set to a range of tolerance of ±0.2 mm. Right side—color map set to a range of tolerance of ±0.5 mm.

A surface-based deviation analysis was carried out using the specific function in the Geomagic Control X software (version 2017.0.0, 3D Systems, Santa Clara, CA, USA). The analysis was complemented by the visualization of the 3D color-coded maps set at two ranges of tolerance, respectively, 0.20 mm and 0.50 mm, to better evaluate and locate the discrepancy between the model surfaces (Figure 4). The maximum deviation calculation was set to 1.00 mm. After the deviation analysis, the percentages of all the distance values within the tolerance range were calculated. The software also allowed for the calculation of the total volume of the 3D models of the mandibular bone; these data were recorded on a spreadsheet and used for comparative analyses.

*Step 4—Matching Percentage Calculation*

Once the deviation analysis was carried out, the percentages (%) of all the distance values were calculated for the two tolerance groups. These values represented the degree of matching between the two models and, thus, show the accuracy of the mandibular models obtained with the tested software (semi-automatic segmentation). 

**Figure 5:**
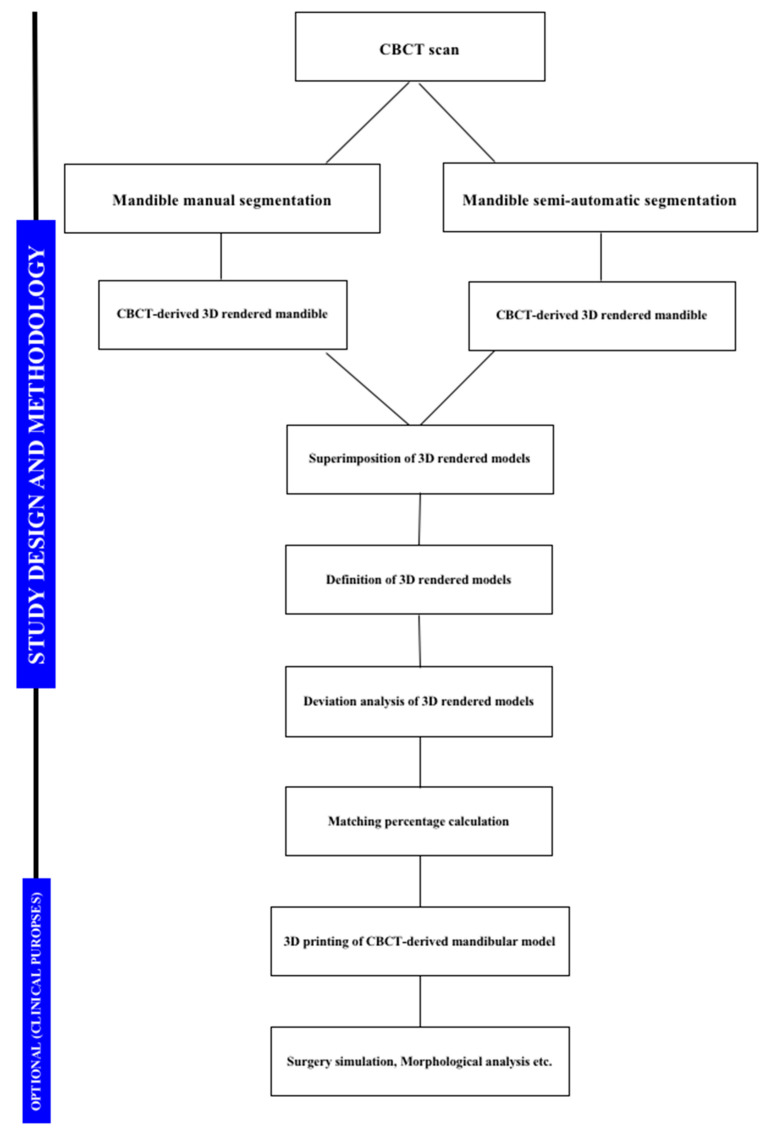
Flow chart of the entire process involved in this study.

The workflow, including segmentation and relative mask generation, was carried out by the same expert operator with 10 years of experience in digital orthodontics (A.L.G.). The images were re-measured 4 weeks after completing the first measurements, and separate spreadsheets were generated in order to blind the operator to the previous data. A flow chart of the entire process is reported in Figure 5. 

*Statistical Analysis*

Differences in the volumetric dimension of the 3D rendered models obtained with each software were tested by using a one-way analysis of variance (ANOVA). The linear regression between the different software was also tested using the manual segmentation as the constant. One-way analysis of variance (ANOVA) was used to assess differences in the matching percentage between the 3D model obtained from manual segmentation (ground truth) and each model obtained from semi-automatic segmentation. Finally, intraclass correlation coefficient (ICC) values were used to check the reliability of the first and second measurements. Data were analyzed using SPSS^®^ version 24 Statistics software (IBM Corporation, 1 New Orchard Road, Armonk, New York, NY, USA) with the significance level set at *p* < 0.05.

## 3. Results

The descriptive characteristics and intraclass correlation coefficients (ICC) for the 3D rendering of the mandible with different software are shown in Table 2. The ICC values ranged from 0.901 to 0.992, thus suggesting that the 3D rendering of the mandible was highly reliable for each software used in this study. 

Small differences were found in the volumetric measurements of mandibular 3D models obtained with different software, ranging from 40.85 cm^3^ (Mimics) to 42.03 cm^3^ (Dolphin 3D); however, such differences were not statistically significant, as reported by the one-way analysis of variance (ANOVA) (Table 3). 

Moreover, high correlations were found between volumetric measurements of the mandible obtained with semi-automatic segmentation (ITK-Snap, Invesalius, Dolphin 3D, Slicer 3D software) and those obtained with the manual segmentation approach (Mimics software), with R coefficients ranging from 0.960 to 0.992. (Table 4). 

According to the one-way analysis of variance (ANOVA) and post-hoc comparison tests, statistically significant differences (*p* < 0.05) were found among the matching percentages (deviation analysis) recorded between the ground truth mandibular shell (manual segmentation) and each mandibular shell obtained from the semi-automatic approach (Table 5). In particular, the mandibular 3D models obtained with ITK-Snap showed the highest matching percentage (Tolerance A = 88.44%, Tolerance B = 97.30%) while those obtained with Dolphin 3D showed the lowest matching percentage (Tolerance A = 60.01%, Tolerance B = 87.76%) (*p* < 0.05). Similar matching percentages were obtained between Invesalius (Tolerance A = 81.79%, Tolerance B = 90.19%) and Slicer 3D (Tolerance A = 82.21%, Tolerance B = 90.57%) (*p* < 0.05). 

## 4. Discussion

In the present study, we investigated the accuracy of the semi-automatic segmentation of the mandible from CBCT scans by comparing the 3D models obtained from different software with a ground truth mandibular model of the same patient obtained from manual segmentation. In this regard, in the absence of a real anatomic structure (dry mandible) or its realistic reproduction (laser scanning), manual segmentation still remains the gold standard for 3D rendering, since it allows for the detection of areas with low bone density or with no well-defined boundaries due to their low contrast and proximity to other structures [3].

In this study, semi-automatic segmentation was performed by using three types of free-source software, Invesalius, ITK-Snap and Slicer 3D, and one commercially licensed software, Dolphin 3D, while manual segmentation was performed by using Mimics software and set as the ground truth model. 

The image scans included in the study were obtained from the same CBCT machine, with the same acquisition parameters. Thus, all factors affecting the accuracy of the 3D model rendering prior to the segmentation process were controlled and limited to the usage of different software [2,17,29]. 

Lastly, we decided to test the mandible for two reasons: (1) the semi-automatic segmentation of the maxilla is notably inaccurate due to variations in cortical bone thickness and the presence of an air-filled structure (sinus), producing bone dehiscence and fenestration artefacts in the 3D model [3,10,30,31]; (2) the 3D reconstruction of the lower jaw has a wider range of clinical applications, especially in orthodontics and maxillofacial fields [3,32,33,34,35,36,37,38].

According to the present findings, the volumetric renderings of the mandible obtained with ITK-Snap, Invesalius and Slicer 3D were notably closer to those obtained with manual segmentation (Mimics), with the 3D rendered model constructed with ITK-Snap and Mimics software being almost identical (mean difference = 0.04 cm^3^, data not shown). Conversely, Dolphin 3D software showed a slight overestimation of the mandibular rendered models with a volumetric difference of about 2 cm^3^ compared with manual segmentation as well as with the other semi-automatic software. However, these differences were not statistically significant. Moreover, all volumetric mandibular measurements obtained with semi-automatic programs showed a high correlation with the manual approach (Mimics), suggesting that all software behaved similarly in defining the mandibular bone contours.

However, volumetric data do not provide a qualitative evaluation of the accuracy of mandibular CBCT-derived models, i.e., precise information of the area of discordance between two surface models. Thus, the accuracy of semi-automatic segmentation was assessed by the superimposition of the 3D models obtained from each investigated software upon those acquired with manual segmentation (same patient). Afterwards, a deviation analysis was used to detect shape differences between the two CBCT-derived mandibular models as well as to obtain precise dimensional information, according to a consolidated methodology [19,20]. The semi-automatic segmentation performed with ITK-Snap, Invesalius, and Slicer 3D showed good accuracy, which is the matching percentage with manual segmentation over 90% (range of tolerance B). The color-coded map showed that the area of mismatch between the ground truth mandibular model (manual segmentation) and the other tested mandibular models (semi-automatic segmentation) was mainly located at the condyle level, with an underestimation of this anatomical region (shown in a turquoise to dark blue color). This is in agreement with previous findings suggesting that the condylar region is difficult to accurately segment due to the lower density of the bone, the overlapping bony structures and the difficulty in separating the condyle with the discus articularis [3,10,35,39,40,41].

The color-coded map showed an overestimation (in the yellow to red fields) of the Dolphin 3D software, which could be responsible for the higher volumetric measurements obtained with this software. Conversely, ITK-Snap, Invesalius, and Slicer 3D reported a slight underestimation (shown in a turquoise to dark blue color) in this area. Such different results could be explained by considering that the proximity of two different structures (roots and cortical bone) with a similar intense radio-opacity may have differently affected the iterative definition of the bony contour during segmentation. In this respect, the resolution of the CBCT scans [3,4] did not adequately allow for the full and accurate automatic distinction of the roots and cortical bone by the different tested software.

With all other variables being equal [2,3,17,21], the most significant factor potentially causing a difference in the measurements on the surface models is the accuracy of the algorithm of the threshold selection. In this respect, the semi-automatic segmentation of the mandible was performed in this study by using the interactive threshold technique, which means that the operator selected the best threshold interval for visualizing the entirety of the anatomic boundaries of the mandible. This process is dependent on the software algorithm, the spatial and contrast resolution of the scan, the thickness and degree of calcification or cortication of the bony structure, and, most importantly, the technical skill of the operator [13]. In this respect, semi-automatic segmentation still relies on the operator’s visual discrimination of the bony structure, with human vision being influenced by lighting conditions, visual acuity fatigue, and grayscale ability [29,35,38,39,40,41,42,43,44]. This is why the segmentation process is still a very subjective method, even with the semi-automatic approach.

However, the tested software featured different semi-automatic segmentation algorithms. In particular, Dolphin 3D features a binary threshold-based volume where the operator uses specific tools to remove the anatomical structures outside the volume of interest; instead, ITK-Snap, Invesalius, and Slicer 3D software were tested using the region-growing algorithm, where the user selects an expanding seed point for the 3D rendering, based on the threshold set. The differences in the interactive rules of the operator may explain some slight differences found in this study, i.e., the overestimation of the rendered volumes with Dolphin 3D software and the underestimation of the rendered volumes with ITK-Snap, Invesalius, and Slicer 3D software. Further studies are required in order to elucidate this aspect.

The accuracy of the segmentation process influences the quality of 3D volumetric representation of craniofacial structures, which is important for the purposes of diagnosis, treatment planning and treatment outcome evaluation [8,16]. Moreover, in modern medicine, 3D printing allows for new, innovative applications such as the construction of patient-specific surgical models and affordable prostheses, as well as more sophisticated procedures, namely bioprinting, tissue engineering and organs 3D printing. In dentistry and maxillofacial fields, the 3D printing of the lower jaw is useful for pre-operative planning or the simulation of surgery involving the three main components of the mandible, i.e., the body, the ramus and the condyle. In this regard, if the digital segmentation of the mandible is not accurate, the physical model obtained with 3D printing will not reliably reproduce the anatomy of the mandible, generating discordance between the treatment plan and the clinical outcomes [45].

Recently, the application of artificial intelligence (AI), through its deep learning paradigm, has shown very promising results in the automated segmentation of anatomical structures from CT and CBCT. In particular, convolutional neural networks (CNNs) have led to a series of breakthroughs in CBCT segmentation [27,30,46,47,48,49,50,51,52], especially when compared to previous methods employing general hand-crafted features, thanks to them being able to learn task-specific features directly from data [49,50,51,52,53,54,55,56,57,58]. Artificial intelligence for craniomaxillofacial structure bone segmentation can overcome two of the primary limits of semi-automatic segmentation, i.e., the operator-dependent and time-consuming process, and future studies are needed for this new scenario.

## 5. Conclusions

According to the present findings, the semi-automatic segmentation of the mandible showed high reliability, as well as high correlation, with the ground truth model (manual segmentation). However, semi-automatic segmentation could cause a slight underestimation of the condylar region of the CBCT-derived 3D mandibular models. In this respect, caution should be taken when evaluating the morphological and dimensional characteristics of the condyles, due to systematic errors in selecting the proper values of the threshold for this complex anatomical region. Consequently, if an accurate definition of condylar boundaries is required, highly skilled clinicians can perform manual refinement. Otherwise, clinicians should refer to companies specialized in 3D imaging technology for this purpose.

## Figures and Tables

**Table 1:** Descriptive analysis and general information of the software tested in the present study.

Name	Version	Developer	Country	Operating System	Type	Segmentation Options
Mimics	21.0	Materialise	Belgium	Windows	Pay per use	Manual and semi-automatic
ITK-Snap	3.8.0	University of Pennsylvania and Utah	USA	Windows, Mac OS X, Linux	Open-source	Manual and semi-automatic
Invesalius	3.0.0	Information Tecnology Center Renato Archer	Brasil	Windows, Mac OS X, Linux	Open-source	Semi-automatic
Dolphi3D	11.0	Patterson Dental Supply	USA	Windows	Pay per use	Semi-automatic
Slicer 3D	4.10.2	Harvard University	United Kingdom	Windows, Mac OS X, Linux	Open-source	Manual and semi-automatic

**Table 2:** Descriptive statistics of intraoperator means and standard deviations (SD) of volumetric measurements of the mandible for each program. Intraclass correlation coefficient (ICC).

EXAMINATIONS	Mimics		ITK-Snap		Invesalius		Dolphin 3D		Slicer 3D	
	Mean	SD	Mean	SD	Mean	SD	Mean	SD	Mean	SD
Measurement 1	40.85	4.16	40.81	4.14	40.04	3.91	42.03	4.07	40.58	4.10
Measurement 2	40.63	4.35	40.43	4.34	40.04	4.09	42.02	4.93	41.08	4.79
ICC	0.992		0.992		0.983		0.938		0.901	

**Table 3:** Comparison among the volumetric measurements of the mandibular models obtained with all the tested software. *p*-value based on one-way ANOVA. Standard deviation (SD); Fisher value (F).

SOFTWARE	Sample	Mean (cm^3^)	SD	Confidential Interval		F	Significance
				Lower Limit	Upper Limit		
Mimics	20	40.85	4.16	38.90	42.80	0.634	NS
ITK-Snap	20	40.81	4.14	38.86	42.74		
Invesalius	20	40.04	3.91	38.21	41.87		
Dolphin 3D	20	42.03	4.07	40.12	43.93		
Slicer 3D	20	40.58	4.10	38.66	42.50		
Total	100	40.71	4.02	39.91	41.50	-	-

**Table 4:** Comparison of segmentation programs with linear regression analysis, using manual segmentation (Mimics) as indipendent variable and semi-automatic segmentation (other software) as dependent variables.

Dependent Variables	Predictor Variables	R	R Squared	Significance	95% Interval Coefficient (B)	
					Lower Limit	Upper Limit
ITK-Snap	Manual Segmentation	0.992	0.983	*p* < 0.001	0.924	1.052
Invesalius		0.960	0.922	*p* < 0.001	0.772	1.033
Dolphin 3D		0.989	0.978	*p* < 0.001	0.894	1.039
Slicer 3D		0.968	0.937	*p* < 0.001	0.831	1.077

**Table 5:** Comparison among the matching percentage between the mandibular models obtained with different semi-automatic segmentation software and the ground truth mandibular model (manual segmentation), according to the deviation analysis. *p*-value based on one-way ANOVA.

Tolerance	Software	Sample	Mean	SD	Confidential Interval		F	Significance
					Lower Limit	Upper Limit		
Tolerance A	ITK-Snap ^a^	20	88.44 ^b,c,d^	1.70	87.65	89.23	405.80	*p* < 0.005
	Invesalius ^b^	20	81.79 ^a,c^	3.10	80.35	83.25		
	Dolphin 3D ^c^	20	60.01 ^a,b,d^	3.10	58.57	61.47		
	Slicer 3D ^d^	20	82.21 ^a,c^	2.90	80.86	83.57		
	Total	80	78.12	11.18	75.63	80.60	-	-
Tolerance B	ITK-Snap ^a^	20	97.30 ^b,c,d^	1.42	96.63	97.97	60.64	*p* < 0.005
	Invesalius ^b^	20	90.19 ^a,c^	3.27	88.66	91.73		
	Dolphin 3D ^c^	20	87.76 ^a,b,d^	2.50	86.60	88.93		
	Slicer 3D ^d^	20	90.57 ^a,c^	1.76	89.75	91.40		
	Total	80	91.46	4.24	90.51	92.40	-	-

^a-b-c-d^ = symbols according to the Scheffè test for post-hoc multiple comparison. SD = Standard deviation. A = range of tolerance set at 0.2 mm; B = range of tolerance set at 0.5 mm.
